# Retrospective Analysis of Potential Adverse Drug Interactions in the Drugs Prescribed to the Elderly at a Tertiary Health Care Center in Raipur, Chhattisgarh, Central India

**DOI:** 10.7759/cureus.53767

**Published:** 2024-02-07

**Authors:** Yazhini Rajendran, Yogendra Keche, Nitin R Gaikwad, Suryaprakash Dhaneria

**Affiliations:** 1 Pharmacology, All India Institute of Medical Sciences, Raipur, Raipur, IND; 2 Pharmacology and Therapeutics, Ruxmaniben Deepchand Gardi Medical College, Ujjain, IND

**Keywords:** mechanism of drug interactions, severity of drug interactions, adverse drug-drug interactions, elderly, polypharmacy

## Abstract

Background

The elderly population differs from adults in having various physiological changes and multiple diseases, which demand the use of multiple medications. The practice of polypharmacy in the elderly leads to numerous harmful effects like adverse drug reactions, adverse drug-drug interactions (DDIs), poor compliance, etc.

Methodology

This study collected 295 case files of elderly patients retrospectively in the Departments of General Medicine, Cardiology and Nephrology after obtaining Institute Ethics Committee approval to look for the potential adverse DDIs with their severity according to the clinical significance.

Results and interpretation

The total number of adverse DDIs identified was 156, the maximum in Category ‘C.’ Salbutamol plus carvedilol/propranolol, ramipril plus telmisartan and ivabradine plus diltiazem were the adverse DDIs categorized under severity ‘X’. The identified DDIs were categorised according to the mechanism such as increased bleeding risk, hypokalemia, hyperkalaemia, reduced effect of drugs and increased effect of drugs.

Conclusion

Polypharmacy can lead to several adverse consequences in the elderly, of which adverse DDIs play a crucial role in harmful health outcomes. This study brings out the significance of predicting drug interactions beforehand which can reduce the risk of bleeding and other risks of hyper/hypokalaemia, hyponatremia and hypoglycaemia.

## Introduction

Ageing is a collective term representing the sum of cumulative local effects at the molecular, cellular and tissue levels [1,2]. The pharmacokinetic and pharmacodynamic parameters change with the increase in age. There is a decrease in absorption due to reduced gastric motility, especially in diabetic elderly patients and an increase in the distribution of lipid-soluble drugs, owing to an increase in total body fat. Concerning pharmacodynamic measures, there may be altered sensitivity of target tissues and altered drug-receptor signal transduction [3]. The blood urea is dependent on protein intake which is reduced in the elderly due to malnourishment. The serum creatinine is dependent on muscle mass which is reduced in the elderly leading to underestimation of the degree of renal failure. Dosing recommendations for the elderly are done using the Cockcroft-Gault formula [4,5].

The elderly population differs from adults in having multiple diseases, disability, and social and behavioural changes. The presence of multiple diseases demands the use of multiple medications in elderly patients, called polypharmacy. It is the concurrent use of five or more medications in the prescription [2,6]. The impact of polypharmacy includes adverse drug reactions, drug-drug or drug-disease interactions, therapeutic duplication, and prolongation of hospitalisation. Medication adherence is also affected by polypharmacy [7,8]. As the number of medications increases, the potential for drug interactions increases [9].

## Materials and methods

This study was carried out as a retrospective analysis of 295 case files of elderly patients admitted between 1st March 2018 and 29th February 2020 in the Departments of General Medicine, Cardiology and Nephrology after obtaining Institute Ethics Committee (IEC) approval with a study duration of one year. A consent waiver for this study was given by the IEC. The sample size was calculated to be 295 by the stratified random sampling technique (including 20% files with missing data) based on the studies to detect potential drug-drug interactions (DDIs) in India where the prevalence was found to be 11%. The in-patient case files of patients aged 60 and above of either sex, admitted in the Departments of General Medicine, Cardiology and Nephrology who were prescribed five or more drugs were included in this study. The case files of elderly patients who were deceased or discharged within 48 hours of hospitalisation, discharged against medical advice, admitted for pure infective aetiology without any co-existing comorbid conditions, admitted in the critical care unit and with incomplete case files were excluded from this study. Medscape Drug Interaction Checker was used for the initial screening of drug interactions [10].

Data collection

The data collected from the case files include patient’s demographic details, diagnosis, clinical findings, and prescription chart in the form of number, dose, route, duration and frequency of drugs prescribed. A total of 2842 case files were screened, of which every tenth case file was selected after applying inclusion and exclusion criteria. The number of case files in General Medicine, Cardiology and Nephrology was 242, 38 and 15, respectively. The adverse DDIs were screened initially using an online tool, Medscape Drug Interaction Checker [10]. The screened DDIs were re-confirmed with standard textbooks like Stockley’s Drug Interactions, Martindale and Goodman and Gillman’s the pharmacological basis of therapeutics and literature to refine the clinically significant ones. The severities of DDIs were checked using UpToDate software [11].

Statistical analysis

Descriptive statistics in the form of proportions were used to represent the data with the help of Microsoft Excel.

## Results

Upon analyzing the data of 295 elderly patients, the majority of the patients were males 186 (63%) and the mean age was 66.10+/-5.99 years. The average number of drugs per prescription was 12.17+/-4.88. The total number of drugs prescribed was 291 (Table 1).

**Table 1:** Demographic data of the case files of patients DDIs: Drug-drug interactions

S.No	Characteristics	Observed Data
1.	Gender	No (%)
	Male	186 (63)
	Female	109 (37)
2.	Age	
	60-69	214 (73)
	70-79	64 (22)
	>=80	17 (5)
	Age (Mean +/- SD)	66.10+/-5.99 years
3.	Average number of drugs per prescription	12.17+/-4.88
4.	Total number of drugs prescribed	291 (11%)
5.	Total number of adverse DDIs identified	156 (16%)

There was a total of 156 adverse DDIs. More DDIs were identified in the General Medicine Department (Table 2); 3133 total drug interactions were initially identified with Medscape Drug Interaction Checker, but only one-third of drug interactions, 999 were found in both Medscape and textbooks or literature (Table 3).

**Table 2:** Number of adverse DDIs in each department DDIs: Drug-drug interactions

S.NO	Adverse Drug-Drug Interactions	Number of adverse drug-drug interactions identified No (%)
1.	General Medicine	113(72)
2.	Cardiology	28(18)
3.	Nephrology	15(10)
	Grand Total	156 (100)

**Table 3:** Total number of drug interactions detected using online tools and textbooks/literature

S.NO	Adverse Drug-Drug Interactions	Medscape drug interactions checker-No (%)	Both Medscape and Textbooks/Literature- No (%)
1.	General Medicine	2631(84)	824(82)
2.	Cardiology	414(13)	154(16)
3.	Nephrology	88(3)	21(2)
		3133 (100)	999 (100)

The maximum number of DDIs belonged to category ‘C severity (72%) followed by 19% in category ‘D’ and 3% in category ‘X’ (Table 4).

**Table 4:** Categorisation of drug interactions as per severity

Severity of Adverse Drug Interactions	Total Number Identified n (%)
X	4(3)
D	26(19)
C	100(72)
B	7(5)
A	1(0.7)

The most common DDI was aspirin and clopidogrel (89(9%)). The adverse DDIs in category X were salbutamol with carvedilol/propranolol and ramipril with telmisartan and ivabradine with diltiazem. The more frequent DDIs in category ‘D’ of severity were aspirin and heparin (5%), heparin and clopidogrel (4%) and sodium bicarbonate and iron compounds (0.9%). Risks of hypotension (4.2%), hyperkalaemia (10.2%), hypokalaemia (8.9%), hypernatremia (0.1%) and hypoglycaemia (1.1%) were identified with DDIs (Table 5).

**Table 5:** Identified drug interactions with their mechanism and clinical significance

Interaction	Drug 1	Drug 2	No.(%) patients	Severity
Bleeding risk	Aspirin	Clopidogrel	89(9)	C
	Heparin	Clopidogrel	41(4)	D
	Aspirin	Heparin	52(5)	D
Deterioration of renal function	Aspirin	Ramipril	47(5)	C
		Enalapril	1(0.1)	C
Hypokalemia	Furosemide	Salbutamol	32(3)	C
		Salmeterol	3(0.3)	C
		Budesonide	29(3)	Not categorised
		Prednisolone	7(0.7)	C
		Hydrocortisone	2(0.2)	Not categorised
		Dexamethasone	2(0.2)	C
	Torsemide	Budesonide	5(0.5)	Not categorised
		Salbutamol	5(0.5)	C
Hyperkalemia	Heparin	Ramipril	31(3)	C
		Enalapril	1(0.1)	C
		Telmisartan	14(1.4)	C
		Olmesartan	2(0.2)	C
	Ramipril	Spironolactone	18(2)	C
		Eplerenone	1(0.1)	C
		Potassium chloride	6(0.6)	C
		Telmisartan	3(0.3)	X
	Spironolactone	Digoxin	8(0.8)	C
		Potassium chloride	6(0.6)	D
		Telmisartan	5(0.5)	C
		Trimethoprim-sulfamethoxazole	2(0.2)	C
	Telmisartan	Potassium chloride	3(0.3)	C
		Digoxin	1(0.1)	C
	Olmesartan	Potassium chloride	1(0.1)	C
	Potassium chloride	Tolvaptan	2(0.2)	Not categorised

Table 5 illustrates the frequency of each DDI with severity. The adverse DDIs were classified according to the mechanism such as bleeding risk, deterioration of renal function, hypokalaemia, hyperkalaemia, hypoglycaemia, hyponatraemia and hypotension. The DDIs with a greater number of frequencies were aspirin+clopidogrel for the risk of bleeding followed by aspirin+metoprolol for the risk of reduced antihypertensive effect. 

There were 18 DDIs not categorized in any of the five classes of severity using UpToDate software but were present in the standard textbooks (Table 6).

**Table 6:** DDIs not mentioned in any of the categories of severity in online software DDIs: Drug-drug interactions

S.no	List of DDIs not included in any of the above-mentioned categories of severity but included in the textbooks
1.	Aspirin and metoprolol
2.	Aspirin and carvedilol
3.	Aspirin and bisoprolol
4.	Aspirin and labetalol
5.	Aspirin and propranolol
6.	Budesonide and furosemide
7.	Budesonide and torsemide
8.	Phenytoin and budesonide
9.	Calcium gluconate and atenolol
10.	Ranolazine and rosuvastatin
11.	Aspirin and chlorthalidone
12.	Clonidine and prazosin
13.	Clonidine and insulin regular
14.	Budesonide and metformin
15.	Hydrocortisone and insulin regular
16.	Hydrocortisone and furosemide
17.	Hydrocortisone and metformin
18.	Potassium chloride and tolvaptan

## Discussion

The total number of adverse DDIs identified was 156, similar to the study conducted by Alkan et al., in which there were 156 (35.1%) drug interactions, of which 4(3%) were category ‘X’, 26 (19%) belong to category ‘D’ and majority (72%) were in ‘C’ [12]. DDIs in category ‘X’ indicate the combination is contraindicated and should be avoided. It is the same compared to the study performed by Sheikh-Taha et al. in which four (3%) DDIs were in the severity ‘X’ though the sample size was large [13]. DDIs in the category ‘D’ means the therapy should be modified since it comes under major severity. It is more compared to the study done by Sheikh-Taha et al. where there were 17 DDIs in category ‘D’. The majority, 100 (72%) were in the severity group ‘C’, requiring monitoring of therapy [13].

The common DDIs were ‘aspirin and clopidogrel’ with the clinical significance mentioned in the MedScape as well as the referred standard textbooks, ‘the concurrent use of more than one antiplatelet drug might increase the risk of bleeding’ in 89(9%) patients and is categorized under ‘C.’ A study by Tillman et al. observed that treatment with clopidogrel plus aspirin increased the risk of major haemorrhage over aspirin alone from 0.2% to 0.9% [14]. Followed by ‘aspirin and metoprolol’ with the justification ‘most NSAIDs decrease the antihypertensive effect, especially in elderly’ in 63 (6%) patients which is not included in any category of severity but present in standard textbooks. It was also identified in a study by Kothari et al. in 21 patients [15]. The next common DDI was ‘pantoprazole and clopidogrel’ in 53 (5%) patients, category ‘C’, for the mechanism ‘clopidogrel efficacy may be reduced by drugs that inhibit CYP2C19.’ The significance mentioned is, ‘the individual patient’s risk factors for gastrointestinal bleeding should be considered against the possible risk of an interaction with clopidogrel, to consider the use of an alternative antiplatelet agent free of interaction with proton pump inhibitors, if their use is essential.’ Lin et al. in their study suggested a recommendation to separate these two drugs with a gap of 12-15 h to prevent any competitive inhibition and suggested taking PPI before breakfast and clopidogrel at bedtime [16].

DDIs in category ‘X’, salbutamol with carvedilol in 6 (0.6%) patients and propranolol in (5(0.5%)) should be avoided as beta-2-agonist and beta blockers antagonise each other. The interaction between short-acting beta-2 agonists and beta blockers is also mentioned in a study on “drug interactions with oral inhaled medications.” by Ajimura et al. [17]. Ramipril and telmisartan combination, which was used in 3(0.3%), should be avoided as there is a risk of dangerous hyperkalaemia. In a study by Rushworth et al., more adverse events like syncope and renal dysfunction occurred with the combination of Ramipril and Telmisartan without an increase in benefit [18]. DDI in ivabradine and diltiazem was seen in two (0.2%) patients; hence, they should not be combined as the metabolism of ivabradine is inhibited by diltiazem, potentially leading to bradycardia and sinus arrest. 'The combination of ivabradine with diltiazem or verapamil (moderate CYP3A4 inhibitors) results in an increase in ivabradine exposure (two to threefold increase in the area under the curve) and an additional heart rate reduction of 5 bpm' as observed in a study by Rushworth et al. [18].

Frequent DDIs in category ‘D’ were aspirin and heparin (5%), heparin and clopidogrel (4%) and sodium bicarbonate and iron compounds (0.9%). In a study by Younossi et al., to compare the effect of combined anticoagulation and low-dose aspirin treatment on upper GI bleeding, upper GI bleeding was more with combined anticoagulation i.e. heparin followed by warfarin in combination with aspirin (20%) compared to the group that received aspirin alone, where no occurrence of upper GI bleeding in any patients [19].

The identified DDIs were classified into various categories like bleeding risk, interactions causing deterioration of renal function, combination causing hypotension, hypoglycaemia, hypokalaemia, hyperkalaemia, one drug reducing the effect of another drug, increasing the effect of the other drug, etc., for better comprehension.

The combination of two drugs increasing the risk of bleeding due to the synergistic effect are aspirin with clopidogrel, heparin with clopidogrel and aspirin with heparin; hence, monitoring of haemoglobin levels or PT/INR or aPTT or bleeding time/clotting time is required [14].

The drugs causing deterioration of renal function are aspirin and ramipril/enalapril because of the reduced synthesis of vasodilating renal prostaglandins by NSAIDs, for which monitoring of renal function test is required [20].

Adverse DDIs causing hypokalaemia (serum potassium <3.5 mEq/L) were the combination of drugs such as furosemide/torsemide with salbutamol/salmeterol/budesonide/prednisolone/ hydrocortisone/dexamethasone which increases serum potassium levels. Hence, monitoring of serum potassium levels is required [21].

Adverse DDIs causing hyperkalaemia (serum potassium >5 mEq/L) were the combinations heparin and ramipril/enalapril/telmisartan/olmesartan; ramipril with spironolactone/eplerenone/potassium chloride/telmisartan; spironolactone and digoxin/potassium chloride/telmisartan/trimethoprim; telmisartan with potassium chloride/digoxin; olmesartan with potassium chloride; potassium chloride with tolvaptan. Hence, monitoring of serum potassium levels is required [15].

There were adverse DDIs that caused hypotension. Due to synergistic effects, there is a risk of acute hypotension and renal insufficiency with ramipril and furosemide/torsemide. The recommendation was to take angiotensin-converting enzyme (ACE) inhibitors at bedtime.

There were adverse DDIs that caused hypoglycaemia. When ramipril is combined with metformin, there is an increased risk of hypoglycaemia. Recovery from hypoglycaemia is delayed when propranolol is combined with insulin. In such cases, normal premonitory signs of a hypoglycaemic attack may not appear, but sweating may be increased. The patients have to be warned of symptoms and signs of hypoglycaemia like palpitations and tremors [21].

There were adverse DDIs that caused hyponatraemia. When combining carbamazepine with hydrochlorothiazide, the risk of hyponatraemia is higher. Thus, monitoring of sodium levels is needed [22].

Adverse DDIs cause reduced effects of the drug. Due to antagonism, the reduced effect of salbutamol/formoterol occurs with carvedilol/propranolol/bisoprolol/metoprolol, potentially precipitating bronchoconstriction [17]. The antihypertensive effect of beta blockers like propranolol/metoprolol/carvedilol/bisoprolol/labetalol can be lost with aspirin requiring frequent monitoring of blood pressure [23]. The diuretic effect of furosemide/torsemide can be decreased with NSAIDs like aspirin/diclofenac/aceclofenac requiring dose adjustment of diuretic. There can be a reduced diuretic effect of spironolactone with aspirin as the active metabolite of spironolactone is blocked by aspirin which requires modification of the antiplatelet drug. Atorvastatin levels could be decreased by combining it with carbamazepine and phenytoin since both induce the metabolism of atorvastatin by CYP3A4. Dose adjustment of statins should be considered. Budesonide levels may get reduced because of CYP3A4 metabolism by phenytoin. The recommendation was to consider corticosteroids for epileptics with proper monitoring. Gastric absorption of furosemide can be reduced with phenytoin needing a furosemide dose increment. Due to the increased metabolism of levothyroxine, the levels of levothyroxine can be reduced with carbamazepine. Hence, monitoring of T3, T4 and TSH is required. Calcium gluconate decreases the effects of atenolol requiring dosage separation. Decreased absorption of iron with pantoprazole/sodium bicarbonate due to reduced gastric acidity; hence the combination could be avoided in anaemic patients.

Adverse DDIs cause reduced anti-hyperglycaemic effects. The anti-hyperglycaemic effect can be reduced with the co-prescription of niacin with metformin/sitagliptin/glimepiride/insulin; prednisolone/hydrocortisone with insulin/glimepiride/metformin, requiring monitoring of blood glucose [24].

Adverse DDIs cause increased effects of the drug. Due to synergism, increased effects of insulin can occur with aspirin, which requires dose adjustment of insulin to prevent hypoglycaemia. When aspirin/aceclofenac is combined with hydrocortisone or dexamethasone or methylprednisolone or prednisolone, the risk of toxic effects like gastrointestinal bleeding and ulceration are more due to synergistic effects [25]. By competing for renal tubular clearance, increased aspirin effect with chlorthalidone could occur. Hence, dose adjustment of diuretics is essential. Increased levels of statins occur with ranolazine by inhibition of P-glycoprotein (MDR1) efflux transporter. Increased levels of atorvastatin and risk of rhabdomyolysis (>1 g/day niacin) are more with atorvastatin and niacin combination. Increased levels of atorvastatin occur by CYP3A4 inhibition by azithromycin. Thus, the patients should be warned regarding the symptoms of myopathy and reporting of muscle pain or weakness. Increased levels of beta-blockers occur with ranolazine by CYP2D6 inhibition. Hence, monitoring of heart rate for the risk of bradycardia is essential. Digoxin levels can increase with amiodarone/ranolazine by inhibiting the P-glycoprotein (MDR1) efflux transporter. Likewise, azithromycin increases the levels of digoxin. A dose reduction of digoxin is needed in such cases. Azithromycin inhibits the P-glycoprotein (MDR1) efflux transporter, thereby increasing amiodarone levels that may lead to an increased risk of Torsades de pointes; thus ECG monitoring is needed [26]. Metoprolol levels rise with amiodarone as it inhibits CYP2D6. Dose adjustment or one drug has to be stopped if the heart rate becomes too low. The potassium loss caused by furosemide/torsemide increases the toxicity of the digitalis [27]. The signs of over-digitalisation, like pulse rate and digoxin levels, have to be monitored if an interaction is suspected. When tramadol and clonazepam/alprazolam/amitriptyline are combined, the risk of sedation is greater leading to falls or accidents. Clarithromycin increases the level of budesonide by CYP3A4 inhibition. A dose reduction of corticosteroids is required. Due to CYP3A4 metabolism, the levels of ivabradine/amiodarone increase with diltiazem. Monitoring of heart rate and ECG are recommended. When heparin and diclofenac are combined, there is an increased effect of anticoagulation, requiring aPTT monitoring.

There are other DDIs apart from the above-mentioned DDIs. Impaired glucose regulation occurs with the octreotide/fluoxetine and insulin/metformin combination. Monitoring of blood glucose levels is required in both situations. Cotrimoxazole increases the level of phenytoin by inhibiting CYP2C9/10. The plasma concentration of phenytoin has to be monitored [28]. Diltiazem increases the level of atorvastatin/ranolazine by CYP3A4 metabolism. The dose of statin has to be reduced and diltiazem should be started with the lowest possible dose and titrated upwards to a maximum of 500 mg twice a day to avoid myopathy and rhabdomyolysis associated with statins. Hypotension risk increases by combining clonidine and prazosin, monitoring of blood pressure is required. Prescribing clonidine with clonazepam increases the risk of sedation. The incidence of skin rashes is higher with the allopurinol and amoxicillin combination, probably due to hyperuricaemia. Itraconazole inhibits the metabolism of nifedipine; hence, blood pressure monitoring is necessary. Cyclosporine level increases with fluconazole as it inhibits CYP3A4 metabolism, requiring dose reduction. The toxic effects of Phenytoin increase with carbamazepine/oxcarbazepine as it inhibits CYP2C19 metabolism. Dose titration of phenytoin is essential. Increased risk of ototoxicity and nephrotoxicity occurs with the combination of tobramycin and furosemide and similarly with amikacin and cyclosporine. Hydroxyzine increases the toxicity of clarithromycin by causing QTc interval prolongation. ECG monitoring is required to avoid cardiac arrhythmia and sudden death. Methotrexate level increases with amoxicillin because of reduced renal clearance. Thus, twice weekly platelet and white cell count for the initial two weeks and folinic acid rescue are needed.

Strengths and limitations

The clinically significant harmful DDIs were identified by both online software and standard textbooks/literature searches. As this was conducted as a retrospective study, immediate feedback could not be dispatched to the treating physicians.

## Conclusions

Adverse DDIs are medication-related adverse events that have hazardous consequences for the health as well as the quality of life of the elderly population. The identified adverse DDIs in this study were 156. The commonly identified DDIs were aspirin with clopidogrel (9%), aspirin with metoprolol (6%) and pantoprazole with clopidogrel (5%). Salbutamol and carvedilol/propranolol, ramipril and telmisartan and ivabradine and diltiazem were the adverse DDIs categorized under severity ‘X’ which should have been avoided. Prediction of drug interactions beforehand can reduce the risk of bleeding and other risks of hyper/hypokalaemia, hyponatremia and hypoglycaemia.
